# Stimulation of CGRP-expressing neurons in the medial cerebellar nucleus induces light and touch sensitivity in mice

**DOI:** 10.1016/j.ynpai.2022.100098

**Published:** 2022-06-23

**Authors:** Mengya Wang, William C. Castonguay, Thomas L. Duong, Michael W. Huebner, Harold C. Flinn, Agatha M. Greenway, Andrew F. Russo, Levi P. Sowers

**Affiliations:** a Department of Neuroscience and Pharmacology, University of Iowa, Iowa City, IA 52242, USA; b Department of Molecular Physiology and Biophysics, University of Iowa, Iowa City, IA 52242, USA; c Center for the Prevention and Treatment of Visual Loss, Veterans Administration Health Center, Iowa City, IA 52246, USA; d Department of Neurology, University of Iowa, Iowa City, IA 52242, USA

**Keywords:** Migraine, Pain, CGRP, Cerebellum, Light aversion, CGRP, calcitonin gene-related peptide, ChR2, channelrhodopsin-2, MN, medial nucleus

## Abstract

Calcitonin gene-related peptide (CGRP) is considered a major player in migraine pathophysiology. However, the location and mechanisms of CGRP actions in migraine are not clearly elucidated. One important question yet to be answered is: Does central CGRP signaling play a role in migraine? One candidate site is the cerebellum, which serves as a sensory and motor integration center and is activated in migraine patients. The cerebellum has the most CGRP binding sites in the central nervous system and a deep cerebellar nucleus, the medial nucleus (MN), expresses CGRP (MN^CGRP^). A previous study demonstrated that CGRP delivery into the cerebellum induced migraine-like behaviors. We hypothesized that stimulation of MN^CGRP^ neurons might induce migraine-like behaviors. To test the hypothesis, we used an optogenetic strategy using *Calca^Cre/+^* mice to drive Cre-dependent expression of channelrhodopsin-2 selectively in CGRP neurons in the cerebellar MN. A battery of behavioral tests was done to assess preclinical behaviors that are surrogates of migraine symptoms, including light aversion, cutaneous allodynia, and spontaneous pain when MN^CGRP^ neurons were optically stimulated. Motor functions were also assessed. Optical stimulation of MN^CGRP^ neurons decreased the time spent in the light, which was coupled to increased time spent resting in the dark, but not the light. These changes were only significant in female mice. Plantar tactile sensitivity was increased in the ipsilateral paws of both sexes, but contralateral paw data were less clear. There was no significant increase in anxiety-like behavior, spontaneous pain (squint), or changes in gait. These discoveries reveal that MN^CGRP^ neurons may contribute to migraine-like sensory hypersensitivity to light and touch.

## Highlights

• CGRP neurons in the medial nucleus of the cerebellum induce migraine-like symptoms.
• Cerebellar CGRP neurons induce photophobia only in female mice, and tactile sensitivity in both sexes.

## 1 Introduction:

Migraine is a neurological disease often described as severe, unilateral pulsating headaches coupled with altered sensory perception, including hypersensitivity to light (photophobia), sound (phonophobia), and touch (cutaneous allodynia). Migraine affects approximately 15% of the global population, women more than men (Stovner et al., 2018), and ranks as the second leading cause of disability worldwide (James, 2018). Approximately 90% of migraine sufferers report photophobia (Russell et al., 1996), which can cause or exacerbate headaches (Noseda and Burstein, 2011), and 25–50% display extracranial cutaneous allodynia (Mathew et al., 2004, Ashkenazi et al., 2007, Burstein et al., 2000, Guy, 2010). Calcitonin gene-related peptide (CGRP) has been established as one of the key players in migraine pathophysiology. Clinical studies have shown that CGRP levels are heightened both during and between migraine attacks (Goadsby et al., 1990, Ashina et al., 2000, Russo, 2019), that peripheral infusion of CGRP prompts the onset of migraine-like headaches in ∼ 66% of migraine patients (Russo, 2019, Ashina et al., 2019), and that CGRP-based drugs that are believed to primarily act in the periphery (Johnson, 2019) are effective in about 50% of patients (Edvinsson et al., 2018, Rapoport and McAllister, 2020). These studies suggest that peripheral actions of CGRP are important in migraine pathophysiology but raise the speculation that CGRP actions in the central nervous system may also contribute to migraine (Russo, 2015, Ho et al., 2010).

Previous animal studies demonstrated that both peripheral (intraperitoneal, i.p.) (Mason et al., 2017) and central (intracerebroventricular, i.c.v.) (Kaiser et al., 2012) administration of CGRP induced similar light-aversive behaviors in wild-type mice. Both behaviors could be attenuated by triptan migraine drugs (Mason et al., 2017, Kaiser et al., 2012). In addition, transgenic mice overexpressing a CGRP receptor subunit in the nervous system displayed light aversion in response to dim light after i.c.v. CGRP injection (Mason et al., 2017, Recober et al., 2010, Recober et al., 2009), as opposed to wild-type mice that had light-aversive behavior only in response to bright light (Kaiser et al., 2012). Light aversion to dim light could also be elicited by direct administration of CGRP into the posterior thalamic nuclei in wild-type C57BL/6J mice, which are a light and pain integration center (Sowers et al., 2020, Noseda et al., 2010). This suggests that heightened sensitivity to CGRP in the nervous system, which is derived from increasing CGRP receptors or applying large amounts of exogenous CGRP, can cause migraine-like light-aversive behavior in mice. These findings further support clinical studies that suggest migraine patients possess a heightened CGRP sensitivity (Russo, 2019, Ashina et al., 2019) and suggest that central CGRP action contributes to migraine-like photophobic behavior in mice.

Analogous to the posterior thalamic nuclei, the cerebellum is an integrative center for sensory and motor inputs (Rondi-Reig et al., 2014, Wiestler et al., 2011). Recent studies have shown that the cerebellum also participates in sensory, emotional, and cognitive aspects of pain, and motor control in response to pain (Moulton, 2010), which are phenotypes exhibited by migraine patients. There are three direct lines of evidence supporting the role of the cerebellum in migraine. First, clinical imaging studies regarding cerebellar activation, structural abnormalities, and functional connectivity changes were reported in migraine patients (Kros et al., 2018). Changes in cerebellar activity and functional connectivity with the thalamus and cortical areas were observed in response to trigeminal stimuli (Mehnert and May, 2019), hinting at a cerebellar role in processing sensory information from the trigeminal system. In addition, upon administration of erenumab, a CGRP receptor antibody, migraine patients exhibited decreased cerebellar activation in response to trigeminal nociceptive stimuli (Ziegeler et al., 2020). It remains unclear whether this is due to secondary to a blockage of peripheral CGRP actions or central actions of the antibody (Ziegeler et al., 2020). Second, vertigo, dizziness and body sway are symptoms observed in migraine patients (Karatas, 2011, Ishizaki et al., 2002), with body sway increasing at higher light intensities (Pinheiro et al., 2020). This movement associated disequilibrium is suggestive of episodic cerebellar dysfunction in migraine patients as the cerebellum plays a key role in motor control. Third, the cerebellum connects to migraine-related regions, including the spinal trigeminal nucleus (Hayashi et al., 1984, Ohya, 1993, Ge et al., 2014) and the thalamus (Baldaçara et al., 2008). But whether these connections involve CGRP is not known. These three lines of evidence highlight the relevance of the cerebellum in migraine pathophysiology.

The cerebellum contains three deep cerebellar nuclei: medial (MN, also known as fastigial nuclei in humans), interposed, and lateral cerebellar nuclei. Which of these three nuclei are associated with migraine pathophysiology? We hypothesized that the MN is a region of interest in migraine pathophysiology due to three lines of evidence. First, the MN receives sensory information from the spinal trigeminal nucleus via the vermis (Ge et al., 2014) and projects to migraine-related brain regions, e.g., the thalamus (Fujita et al., 2020). Second, pain-related responses were decreased in response to visceral stimuli upon injection of glutamate or a glutamate receptor agonist into the MN (Saab and Willis, 2002, Zhen et al., 2018). Finally, CGRP and its receptor components are expressed in the MN (Warfvinge and Edvinsson, 2019). These findings suggest that the MN, specifically CGRP and its receptors in the MN, may be associated with migraine pathophysiology. A previous study has demonstrated that CGRP injections into the MN induced migraine-like behaviors, including light aversion, anxiety-like, cutaneous allodynia and nociceptive squinting behaviors (Wang et al., 2022). Due to the CGRP distribution in the MN, we pursued the idea of the CGRP-expressing neurons of the MN (MN^CGRP^) being involved in migraine pathophysiology.

To assess how these MN^CGRP^ neurons contributed to migraine-like behaviors, we used genetically engineered *Calca^Cre/+^* mice, where Cre recombinase is inserted into *Calca*, the gene encoding α-CGRP (Carter et al., 2013, Amara et al., 1982). Using an optogenetic strategy and Cre-dependent channelrhodopsin-2 (ChR2) expression, we selectively activated MN^CGRP^ neurons and performed a battery of tests to assess preclinical behaviors that are surrogates of migraine-like symptoms and to assess motor function. The results demonstrate that optical stimulation of MN^CGRP^ neurons evoked light aversion only in female mice without accompanying anxiety, and tactile hypersensitivity without spontaneous pain or gait alterations.

## 2 Materials and methods

### Animals

*Calca^Cre/+^* mice were kindly provided by R. Palmiter (Carter et al., 2013). These mice have Cre inserted into exon 2 of the *Calca* gene. Mice were aged between 10 and 21 weeks when the surgery began. A total of 47 *Calca^Cre/+^* mice (24 females; 23 males) were used for this study. Female mice had an average starting body weight of ∼ 20–25 g and males were ∼ 26–30 g. All animals were housed on a 12-hour light cycle with access to water and food ad libitum. Animal procedures followed the ARRIVE guidelines and were approved by the Iowa City Veterans Administration and University of Iowa Animal Care and Use Committees and performed in accordance with the standards set by the National Institutes of Health.

### 2.2 Virus and stereotaxic surgery

Stereotaxic surgery for virus injection and optical fibers into the MN of the right cerebellum was performed under isoflurane anesthesia (induction 5%, maintenance 1.5%–2%). AAV2-EF1a-DIO-ChR2(E123A)-mCherry or the vector control AAV2-EF1a-DIO-mCherry (200 nl) from UNC Vector Core was injected into the right MN at the rate of 100 nl/min for 2 min. AAV2-EF1a-DIO-EYFP was also used but only for the validation of CGRP expression in the MN and fibers from the MN. The stereotaxic coordinates used for injecting the MN are: anterior/posterior (AP), −6.5 mm posterior to bregma; medial/lateral (ML), −0.85 mm lateral to the midline; and dorsal/ventral (DV), −2.7 mm ventral to the pial surface according to the coronal Allen Brain Reference Atlas. Following virus injection, an optical fiber (4 or 4.5 mm in length, a core diameter of 200 μm, an outer diameter of 240 μm, a numerical aperture of 0.22, Doric Lenses) was implanted 0.4 mm dorsal to the injection coordinates for the MN. The optical fiber was secured with bone anchor screws (Stoelting), adhesive (Pacer Technology), and dental cement (Stoelting). Behavioral experiments were performed at least 3 weeks after surgery to allow for adequate viral transduction.

### 2.3 Behavioral tests

#### 2.3.1 Light/dark assay

The testing chamber was a transparent, seamless open field chamber (Med Associates) divided into two zones of equal size by a black infrared-transparent dark insert. The dark insert was customized based on the original dark insert from Med Associates. Two modifications were made (Sowers et al., 2020, Wang et al., 2021) to help avoid any interference with mouse movement when a mouse is connected to a fiber-optic patch cord. The top of the dark insert was extended over the light area as a triangular porch (H = 6.4 cm) with a hole (Inner diameter = 2.60 cm) for embedding a rotary joint. The opening of the dark insert was 6.10 × 5.08 cm (W X H) with a small slit (0.95 × 10.16 cm in W X H) between the top and the opening of the dark insert. The mouse activity was collected with infrared beam tracking and Activity Monitor software (Med Associates), as previously described (Kaiser et al., 2012, Wang et al., 2021). Mice were tested without pre-exposure to the chamber at the light intensity at 2.7 × 104 lx. The light allowed into the back-right corner of the dark chamber is ∼ 40 lx.

Mice were placed in the light zone of the light/dark chamber and data were collected for 40 min and analyzed in sequential 5 min intervals. During the assay, mice were optically stimulated at 20 Hz with a 5 ms pulse width during twenty 1 min intervals, each preceded by 1 min without stimulation using 10 mW power at the optical probe tip from a diode-pumped, solid-state laser (473 nm, 100 mW, OptoEngine LLC). Light aversion was expressed as both a function of time over the 40-min testing period and the average time in light for individual mice per 5 min interval. Motility outcomes were collected during the light/dark assay, as described previously (Kaiser et al., 2012, Wang et al., 2021). Briefly, resting time was measured as the percentage of time mice did not break any new beams in each zone normalized against time spent in the respective zone.

#### 2.3.2 Open field assay

This assay is to measure anxiety-like behavior. The apparatus was the same as in the light/dark assay with the absence of the dark insert and in the open with room lighting (∼1000 lx) to allow the mouse to move freely when connected to a fiber-optic patch cord, as described previously (Sowers et al., 2020, Wang et al., 2021). Mice were placed in the middle of the open field chamber with the same stimulation pattern as in the light/dark assay (10 mW power at the optical probe tip, 20 Hz, 5 ms pulse width, alternating 1 min on/off epochs starting with the laser being off for the first min). The periphery from the border was measured as 3.97 cm, leaving the center with an area of 19.05 × 19.05 cm. To calculate the percentage of time the mouse spent in the center, the time in the center was divided by the total time in the chamber.

#### 2.3.3 Von Frey test

The test is to evaluate the mechanical nociceptive threshold. After habituation to the testing room for one hour, investigators gently restrained the mouse and connected the optical fiber on the mouse head to a fiber-optic patch cord. Next, the mouse was placed in an acrylic chamber (10.80 × 6.99 × 14.61 cm in W × D × H) over a grid support (Bioseb, France). When the mouse was standing on all four paws, calm and still, von Frey filaments were applied to the right or left hind paws without optical stimulation (baseline) or with optical stimulation. Mice were allowed to rest in the von Frey chambers for ∼ 15 min or their home cages for ∼ 60 min between the baseline and stimulation measurements.

To exercise research rigor and ensure reproducibility, the investigator who applied filaments was blinded to the viral variants. The test was based on the up-and-down method as previously described (Dixon, 1965, Chaplan et al., 1994). A set of 8 von Frey filaments was used from A (0.008 g) to H (1 g) (Bioseb, France). Filaments were applied for 5 s to the skin of the mouse plantar surface of the hind paw, with D (0.07 g) as the starting filament. A withdrawal response was considered when mice withdrew, shook, or licked the tested hind paw. If a withdrawal response was observed with a particular force of filament, then a lower filament force was used. On the other hand, if a withdrawal response was not observed, then a higher filament force was used. This method of monitoring withdrawal response was used for 5 applications of filaments after the first change in pattern was assessed. The responses can then be recorded and calculated to determine the withdrawal threshold at which 50% of mice withdrew their hind paws using an established equation (Dixon, 1965, Chaplan et al., 1994). However, the threshold data produced in this method are not continuous and cannot be analyzed using parametric statistics. Thus, to obtain normal distribution, the 50% thresholds (g) were transformed into log format for data analysis and figure plotting unless otherwise indicated.

The optical stimulation pattern in the von Frey test was adjusted from the light/dark assay since we tried to investigate the effect of optically stimulating MN^CGRP^ neurons on the tactile sensitivity simultaneously and the completion of performance of up-and-down method usually takes 30 s–3 min while the light/dark assay is lasting 40 min. Accordingly, filaments were applied upon the start of the optical stimulation. The stimulation length was dependent on responses to filaments of individual mice, approximately at a range of 30 s–3 min, with the maximum of 5 min, and the stimulation is at 20 Hz continuously instead of alternating 1 min on/off epochs. The stimulation pattern is: 10 mW power at the optical probe tip, continuous 20 Hz pulses with 5 ms pulse width.

#### 2.3.4 Automated squint assay

This assay is to evaluate spontaneous pain by measuring the right-eye pixel areas recorded by a camera. Mice were acclimated to a customized collar restraint to reduce stress induced by the restraint as well as struggle or head movement as described previously (Rea et al., 2018, Rea et al., 2021). Mice underwent acclimation for 20 min per session for three sessions. On the test day, after habituation to the testing room for one hour, the mouse was placed in the restraint. Squint was recorded for 3 min to establish the baseline, and subsequently recorded for another 3 min with optical stimulation. The stimulation pattern in the automated squint assay was adjusted from the light/dark assay because of the possible stress induced by restraint when mice were restrained for a long period of time. Like the von Frey test with a slight change in stimulation pattern, we performed laser stimulation at 20 Hz with a pulse width of 5 ms for 3 min continuously.

For CGRP treatment experiments, rat α-CGRP (Sigma-Aldrich) diluted in 1X phosphate-buffered saline (PBS; HyClone™) was used. The squint was recorded for 3 min before the treatment as the baseline. Next, CGRP (i.p., 0.1 mg/kg) was administered to the mice and subsequently returned to the home cage to rest for 30 min. After 30 min, the mouse was placed in a restraint and underwent squint recording for 3 min as the treatment recording, immediately followed by another squint recording for 3 min with optical stimulation (20 Hz, 5 ms pulse width for 3 min) as “treatment and stimulation recording”. These mice were connected to the fiber-optic patch throughout the habituation phase to make mice habituate to the optic-fiber patch, and the recording phases (baseline, stimulation, treatment, and “treatment and stimulation” recordings) to allow for consistency. The recordings were performed under room light.

Pixel area measurement for the right eye was calculated every 0.1 s (10 frames/s) in the recordings using trained facial detection software (FaceX, LLC, Iowa City, IA) with the resulting values compiled with a custom MATLAB script. Individual frames containing a tracking error rate of greater than 15% were excluded.

#### 2.3.5 Gait dynamic assay

DigiGait imaging system (Mouse Specifics Inc, Boston, MA, USA) was used to assess gait dynamics. This system is composed of a transparent chamber (17.14 × 5.08 × 15.24 cm in W × D × H), a transparent plastic treadmill belt, an under-mounted digital camera, a light over the chamber for camera capturing videos (∼7200 lx), software to record videos (DigiGait Imager), and an image analysis software (DigiGait Analysis).

Mice underwent habituation to the testing room for one hour prior to starting the assay. Investigators gently restrained the mouse and connected the optical fiber on mouse head to a fiber-optic patch cord. The mouse was placed in the transparent chamber for 1 min to allow them to explore the chamber. The treadmill belt was subsequently turned on and the mouse was run at 16 cm/s. Images of the paws were ventrally captured during the run using the under mounted digital camera. Each mouse ran until roughly 3–5 s of continuous gait was observed, a range sufficient to acquire adequate quantification of gait parameters. Mice underwent recordings without optical stimulation as the baseline, or with optical stimulation (10 mW power at the optical probe tip, continuous 20 Hz pulses with 5 ms pulse width).

DigiGait capture process is usually short – the mouse gets tired after running for a while. Therefore, a clip of roughly 3–5 s with continuous running was chosen and analyzed out of approximately 10–60 s of total running. When analyzing the relationship between the paw withdrawal threshold and the stimulation interval, we found that the maximum response occurred at approximately 50–90 s (data not shown). To achieve the possible maximum responses, the laser was turned on 10 s after the mouse was placed in the DigiGait chamber. After 50 s, the treadmill was turned on, and mouse gait data collection was initiated. The laser was on until the desired continuous running was obtained. The stimulation length was approximately at a range of 60–110 s. Between the baseline and stimulation runs, a minimum of one-hour rest in the home cage for each test subject was allotted to allow mice to recover from the previous running.

The mouse paw prints were analyzed by DigiGait Analysis to identify stride length and frequency. A complete stride was defined as the portion of foot strike to subsequent foot strike on the treadmill belt of the same foot.

### 2.4 Histology

After finishing all the behavioral tests, mice were sacrificed, and the accuracy of viral targeting and fiber optic probe tip placement were assessed histologically. Specifically, mice were deeply anesthetized with ketamine/xylazine (87.5 mg/kg/12.5 mg/kg, i.p,). Then the mice were perfused transcardially with 1X PBS and subsequently with 4% paraformaldehyde. Brains were extracted and post-fixed in 4% paraformaldehyde at 4 °C overnight, followed by sequential equilibration in 10, 20, and 30% sucrose for 24 h per concentration at 4 °C. Brains were embedded in a tissue-freezing medium and stored at − 80 °C until use. 40 μm coronal slices were collected and mounted onto Superfrost Plus slides (Fisher Scientific) using antifade mountant with DAPI (VECTASHIELD). Images were captured using a confocal microscope (Zeiss Corporation, LSM 880) or a scanning microscope (Olympus, VS120).

The immunohistochemistry experiment was performed as previously described (Sowers et al., 2020). Briefly, the free-floating sections were rinsed 4 times in 0.1 M phosphate buffer (PB) (0.08 M Na_2_HPO_4_, 0.02 M NaH_2_PO_4_, pH 7.4) for 5 min each at room temperature. Sections were then incubated in a blocking solution composed of 0.1 M PB with 10% goat serum (Sigma, G9023) and 0.3% Triton-X-100 for 1 h at 4 °C. Subsequently, sections were incubated with rabbit anti-CGRP antibody (1:1000, Sigma, C8198) at 4 °C overnight. After 3 washes in 0.1 M PB with 10% goat serum and 0.3% Triton-X-100 for 5 min each, sections were incubated with goat anti-rabbit Alexa 405 (1:1000, Thermo Scientific, A31556) for 1 h at room temperature in the dark. Following staining, sections were rinsed twice in 0.1 M PB with 10% goat serum and 0.3% Triton-X-100 for 5 min per rinse, then one time in 0.05 M PB for 5 min. Finally, sections were counterstained by incubation with TO-PRO-3 iodide and were mounted onto Superfrost Plus slides (Fisher Scientific) using antifade mountant (VECTASHIELD). Images of tissue sections were captured using a Leica Y15P confocal microscope.

### 2.5 Experimental design

Behavioral experiments were conducted between the hours of 7:00 A.M. and 6:00 P.M., and mice were habituated to the behavioral testing room for one hour prior to commencing experiments. Mice were allowed to recover in their home cages for at least one day between each behavioral test. The light/dark assay was conducted first followed by the open field assay. Subsequently, the von Frey test and the gait dynamic assay were performed. The automated squint assay was done last to avoid disturbance to other assays coming from the possible stress induced by restraint.

### 2.6 Statistical analysis

The projected sample size estimated to be required for the study was determined using a power analysis based on previous studies from the lab using ClinCalc.com. An alpha of 0.05 and a power of 0.80 was used. The analysis determined a projected sample size of 10 mice in each group to ensure the experiment was sufficiently powered. Data were analyzed using GraphPad Prism 9 and are reported in Supplementary Table 1. Significance was set at P less than 0.05. Error bars represent ± SEM.

A two-way repeated measure analysis of variance (ANOVA) was performed when data were plotted as a function of time in the light/dark and open field assays (factor: treatment and time). When the interaction or the treatment was significant, Šídák's multiple comparisons test was used as the post-hoc analysis. An unpaired *t*-test was performed for bar graphs with scatter points to compare the effect of each treatment. When comparing female and male ChR2 groups, the delta was calculated by subtracting the values of individual ChR2 mice from the mean of the respective mCherry group, and then was analyzed using an unpaired *t*-test.

When data were plotted for the scatter plot graphs of the von Frey, gait dynamic and squint experiments (factor: treatment and condition), a two-way repeated measure ANOVA was performed. For plantar von Frey, automated squint with optical stimulation only, and gait dynamic assays, when the interaction or the condition was significant, a post-hoc paired *t*-test was used to compare between the baseline and the stimulation. It should be noted that the post-hoc paired *t*-test was not corrected for multiple comparisons. When comparing withdrawal thresholds between mCherry and ChR2 groups, the 50% threshold (g) values for baselines were subtracted from the stimulation measurements for each group, and were then analyzed using an unpaired *t*-test. When analyzing the sex difference in the withdrawal threshold, changes of 50% thresholds (g) in the ChR2 group in each sex by extracting the respective baseline from the stimulation measurements were compared using an unpaired *t*-test. For the automated squint assay with optical stimulation and CGRP treatment, after achieving the significance in the interaction or the condition from a two-way repeated measure ANOVA, a one-way repeated measure ANOVA was performed due to 3 groups. If there is a significance, Tukey's multiple comparisons test was used to compare the effect between baseline and treatment with/without stimulation.

A total of 3 mice died due to the surgical procedure or lost the optical fibers before running any behavioral test. 3 mice were excluded because the optical probe was off-target or no mCherry expression was found in the MN. One mouse brain was missing before targeting evaluation, so this mouse was excluded. One mouse from the light/dark assay and the open field assay was excluded due to a chamber recording problem. In the von Frey test, one mouse was excluded due to a laser issue. In the gait dynamic assay, 7 mice in total were excluded with 2 due to the loss of optic fibers and 5 due to video recording problems. In the automated squint assay with optical stimulation only, 2 mice were excluded due to the loss of the optic fibers. In the automated squint assay with optical stimulation and i.p. 0.01 mg/kg CGRP treatment, 3 mice were excluded due to laser or video recording problems. In the automated squint assay with optical stimulation and i.p. 0.1 mg/kg CGRP treatment, 3 mice were excluded due to laser or video recording problems and optic fiber loss. Mouse numbers used for each experiment are reported in the figure legends.

## 3 Results

### 3.1 Validation of CGRP expression and projections in the MN of heterozygous *Calca^Cre/+^* mice

**Fig. 1:**
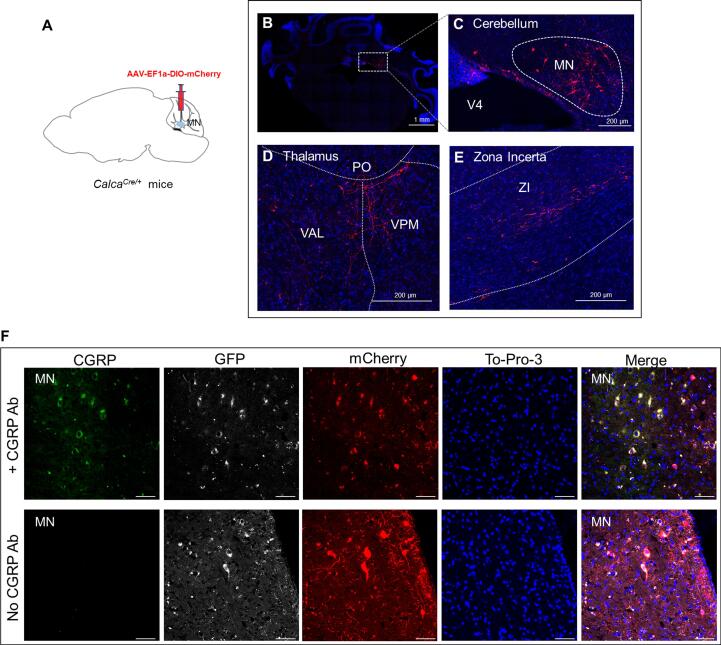
**Validation of CGRP expression and projections in the MN of *Calca^Cre/+^* mice.** (A) Schematic of the injection of Cre-dependent virus AAV-EF1a-DIO-mCherry into the MN of *Calca^Cre/+^* mice. (B) Representative example of a mouse with the expression of mCherry (red) in the MN. (C) A magnified image of the area within the rectangle in (B) showing mCherry-positive cell bodies and fibers in the MN (red, mCherry; blue, DAPI). (D and E) Representative examples showing fiber projections from the same mouse shown in (B). The mCherry signals were found as fibers in the contralateral posterior thalamic nuclei (D) and zona incerta (E). (F) Representative sections of the MN from a *Calca^Cre/+^* mouse injected with AAV-EF1a-DIO-mCherry into the MN and stained for CGRP (green). Scale bar = 50 µm. MN: medial cerebellar nucleus; PO: posterior complex of the thalamus; V4: fourth ventricle; VAL: ventral anterior-lateral complex of the thalamus; VPM: ventral posteromedial nucleus of the thalamus; ZI: zona incerta.

CGRP is reported to be in the MN of rat brains (Warfvinge and Edvinsson, 2019). However, to our knowledge, no previous studies have reported CGRP in the mouse MN. To investigate CGRP expression in the mouse MN, we used *Calca^Cre/+^* mice, in which the Cre recombinase gene was inserted into the exon 2 of the *Calca* gene, which encodes α-CGRP (Carter et al., 2013, Amara et al., 1982). Heterozygous *Calca^Cre/+^* mice were injected with a Cre-dependent virus carrying an mCherry reporter (AAV2-EF1a-DIO-mCherry) (Fig. 1A). Post-hoc analyses found mCherry signals in the MN (Fig. 1B and C). In addition, we observed mCherry-positive fibers in contralateral posterior thalamic nuclei and zona incerta (Fig. 1D and E). This finding indicates that MN^CGRP^ neurons project to the contralateral thalamus and zona incerta. We also used another viral vector AAV2-EF1a-DIO-EYFP and found that the results were similar, validating CGRP expression in the MN and projections from MN to posterior thalamic nuclei and zona incerta (Supplementary Fig. 1).

CGRP immunostaining revealed clear immunoreactive signals within the MN (Fig. 1F). The CGRP staining was colocalized with mCherry reporter expression, as well as with GFP tag from the Cre gene insertion (Fig. 1F). Thus, injection of a Cre-dependent virus containing an mCherry reporter into the MN of *Calca^Cre/+^* mice resulted in specific expression of mCherry in CGRP-positive neurons in the MN.

### 3.2 Optical stimulation of MN^CGRP^ neurons induced light-aversive behavior only in female mice

**Fig. 2:**
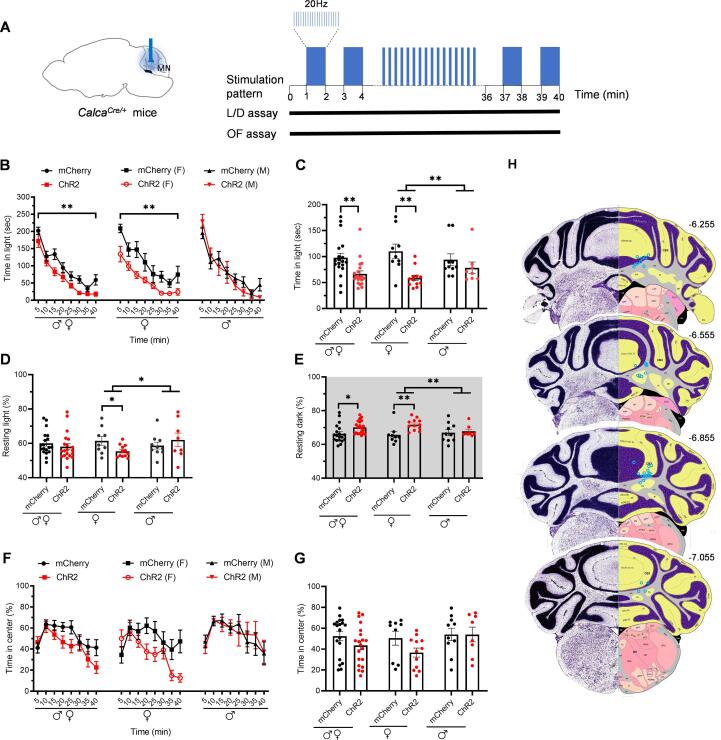
**Optical stimulation of MN^CGRP^ neurons induced light-aversive behavior only in female mice.** (A) Diagram representing the optogenetic stimulation strategy in the MN in *Calca^Cre/+^* mice during light/dark (L/D) or open field (OF) assays. Left panel: Cre-dependent virus encoding either ChR2 or mCherry (control) was injected in the MN and the optical fibers were implanted above the MN. Right panel: optogenetic stimulation parameters. Mice were optically stimulated at 20 Hz with a 5 ms pulse width during twenty 1 min intervals, each preceded by 1 min without stimulation during the behavioral assays. (B) Time in light every 5-min block during 40-min light/dark assay with the optical stimulation of *Calca^Cre/+^* mice. Time in light for all mice (left panel) (mCherry: n = 19; ChR2: n = 20), female mice (middle panel) (mCherry: n = 9; ChR2: n = 12), and male mice (right panel) (mCherry: n = 10; ChR2: n = 8). All mice in B were further analyzed in C-E. (C) Mean time in light per 5-min block for individual mice from B. (D and E) Mean percentage of time spent resting in the light (D) and the dark (E) zones per 5-min block for individual mice. All mice (left panel), female mice (middle panel), and male mice (right panel). (F) Percentage of time spent in the center of the open field every 5-min block during 40-min testing period with optical stimulation of *Calca^Cre/+^* mice. Time in center for all mice (left panel) (mCherry: n = 19; ChR2: n = 20), female mice (middle panel) (mCherry: n = 9; ChR2: n = 12), and male mice (right panel) (mCherry: n = 10; ChR2: n = 8). Data are from two independent experiments. All mice in F are further analyzed in G. (G) Mean percentage of time in the center per 5-min block for individual mice from F. (H) Schematic of positions of optical fiber tips superimposed on Allen Mouse Brain Atlas coronal images. Numbers indicate the distance (mm) from bregma in the anteroposterior plane. Data are from two independent experiments. For all panels, data are the mean ± SEM. Statistics are described in Supplementary Table 1. *P ≤ 0.05, **P ≤ 0.01.

We next sought to investigate if MN^CGRP^ neurons mediate light-aversive behavior. A Cre-dependent AAV ChR2 vector (AAV2-EF1a-DIO-hChR2(E123A)-mCherry) or a control vector lacking ChR2 (AAV2-EF1a-DIO-mCherry) was injected into the right MN of *Calca^Cre/+^* mice. At least three weeks after virus injection, mice were optically stimulated during a 40 min-light/dark testing period (20 Hz, 5 ms pulse width, alternating 1 min off/on epochs, Fig. 2A).

When stimulated, mice expressing ChR2 in MN^CGRP^ neurons spent less time in the light zone compared to control mCherry-expressing mice (Fig. 2B). When separated by sex, there was a significant decrease in the time in light in female mice (Fig. 2B), while no difference was observed in male mice (Fig. 2B). On average, the female mCherry-expressing mice spent 110 s in the light per 5-min interval, and female ChR2-expressing mice spent 59 s (Fig. 2C). There was also a significant difference between female and male ChR2 groups (Fig. 2C). This indicates that optical stimulation of MN^CGRP^ neurons induces light-aversive behavior only in female mice.

Resting behavior in light and dark zones with optical stimulation was evaluated in the same light/dark assay. The percent resting time in the light zone was decreased and the percent resting time in the dark zone was increased in female ChR2-injected mice compared to the female mCherry group (Fig. 2D and E; Supplementary Fig. 2). No difference was observed in the percent resting time in the light or dark zones in male groups (Fig. 2D and E). When comparing male and female ChR2 groups, significant differences were detected in resting time both in the light and dark (Fig. 2D and E).

To distinguish whether the behaviors in the light/dark assay were due to light aversion or an increase in anxiety, a light-independent open field anxiety assay was conducted with the same optogenetic stimulation pattern as in the light/dark assay (Fig. 2A). All mice spent similar amounts of time in the center regardless of viral vector or sex although there was a trend in the female group to spend less time in the center (Fig. 2F and G). We performed a post-hoc power analysis to estimate how many female mice might be needed to reach a power of 80%, and found it requires more than twice the number of current female mice. This finding indicates that optical stimulation of MN^CGRP^ neurons did not induce light-independent anxiety-like behavior. Furthermore, it suggests that light aversion detected in female mice is not driven by anxiety alone.

For all mice, post-hoc confirmation of the targeting site was performed. Sites of optical probe tips are shown in Fig. 2H. As mentioned in Methods, 3 mice were excluded from the behavioral data because of the off-target probe placement or undetectable mCherry expression in the MN, and the probe tips for these 3 mice are not shown in Fig. 2H.

### 3.3 Optical stimulation of MN^CGRP^ neurons induced plantar tactile hypersensitivity

**Fig. 3:**
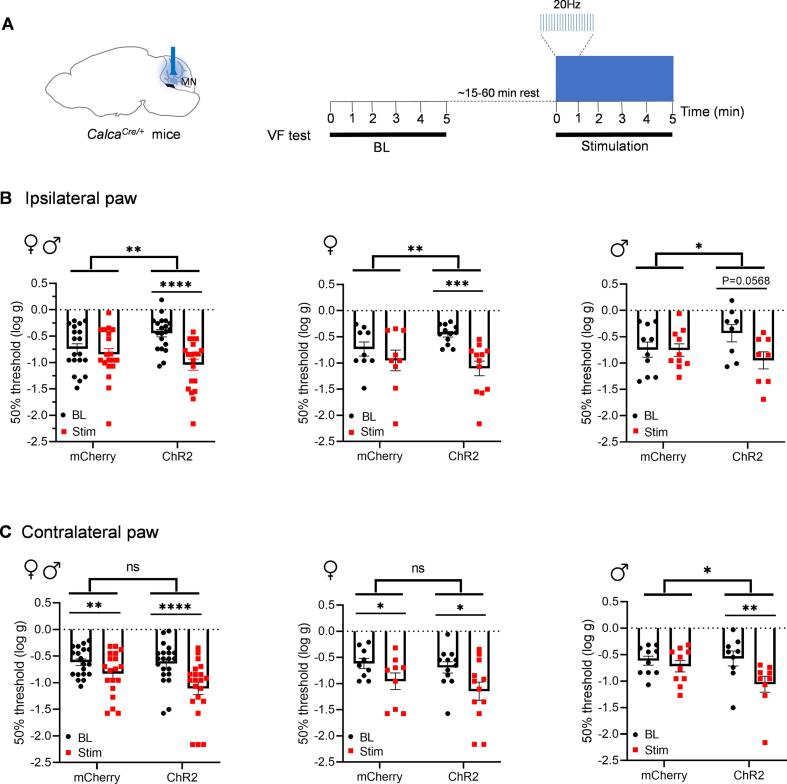
**Optical stimulation of MN^CGRP^ neurons induced plantar tactile hypersensitivity.** (A) Diagram representing the optogenetic stimulation strategy in the MN in *Calca^Cre/+^* mice during the von Frey test. Left panel: cre-dependent virus encoding either ChR2 or mCherry (control) was injected in the MN and the optical fibers were implanted above the MN. Right panel: experimental design. Baseline (BL) was first measured without stimulation. Next, after approximately 15–60 min resting, mice were optically stimulated at 20 Hz with a 5 ms pulse width upon filament applications and until the completion of von Frey up-and-down method, approximately at a range of 30 s-3 min, with the maximum of 5 min. (B) Plantar tactile hypersensitivity was assessed without (as baseline) and with stimulation in *Calca^Cre/+^* mice injected with AAV encoding either ChR2 or mCherry. The individual thresholds of right hind paws for all mice (left panel) (mCherry: n = 19; ChR2: n = 20), female mice (middle panel) (mCherry: n = 9; ChR2: n = 12), and male mice (right panel) (mCherry: n = 10; ChR2: n = 8). (C) The individual thresholds of left hind paws for all mice (left panel) (mCherry: n = 19; ChR2: n = 21), female mice (middle panel) (mCherry: n = 9; ChR2: n = 12), and male mice (right panel) (mCherry: n = 10; ChR2: n = 9). Data are from two independent experiments. For all panels, data are the mean ± SEM. Statistics are described in Supplementary Table 1. *P ≤ 0.05, **P ≤ 0.01, ***P ≤ 0.001, ****P ≤ 0.0001.

Cutaneous allodynia is reported by about 60% of migraine patients (Lipton et al., 2008), and furthermore in 25–50% of patients, the allodynia is extracephalic (Ashkenazi et al., 2007, Burstein et al., 2000, Guy, 2010, Mathew et al., 2004). Thus, we wanted to explore the role of MN^CGRP^ neurons in the tactile sensitivity of the plantar hind paw. *Calca^Cre/+^* mice injected with ChR2 or control viral vectors in the right MN were optically stimulated during the plantar von Frey test (10 mW power at the optical probe tip, 20 Hz, 5 ms pulse width, Fig. 3A). Mice expressing ChR2 in the MN^CGRP^ neurons showed a significant decrease in the ipsilateral right paw withdrawal threshold with optical stimulation compared to their respective baselines (Fig. 3B, left panel). No difference was observed in control mCherry-expressing mice between stimulation and the baseline conditions (Fig. 3B, left panel). Likewise, a comparison of the deltas between baseline and stimulated values of the mCherry control with the ChR2 experimental groups revealed a significant difference (Fig. 3B, left panel). When data were separated by sex, the female ChR2 group with stimulation showed a significant decrease in the right paw withdrawal threshold compared to the respective baseline, and there was a trend for the male ChR2 group (Fig. 3B, middle and right panels). Changes in withdrawal thresholds from the baseline to the stimulation between mCherry and ChR2 groups were significant for both female and male mice (Fig. 3B, middle and right panels). There was no sex difference in the threshold change in ChR2 groups (Supplementary Table 1).

In the contralateral left paw, results were more complicated due to a significant decrease in withdrawal threshold with stimulation compared to baselines in not only the ChR2 group but also the control mCherry group (Fig. 3C left panel). This was driven primarily by the female mice (Fig. 3C, middle panel). Consequently, a conclusion cannot be drawn from the female contralateral paw data. In contrast, a significant reduction in the withdrawal threshold was observed in male mice expressing ChR2 in MN^CGRP^ neurons with optical stimulation compared to the baseline (Fig. 3C, right panel). Male mice also exhibit significant changes in withdrawal thresholds from the baseline to the stimulation between mCherry and ChR2 groups (Fig. 3C, right panel). No sex difference in the threshold change in ChR2 groups was detected (Supplementary Table 1). Altogether, these data suggest that optical stimulation of MN^CGRP^ neurons induces cutaneous allodynia in ipsilateral hind paws in both sexes. While the data are less clear for the contralateral paws, cutaneous allodynia is apparent at least in male mice.

### 3.4 Optical stimulation of MN^CGRP^ neurons did not induce nociceptive squinting behavior

**Fig. 4:**
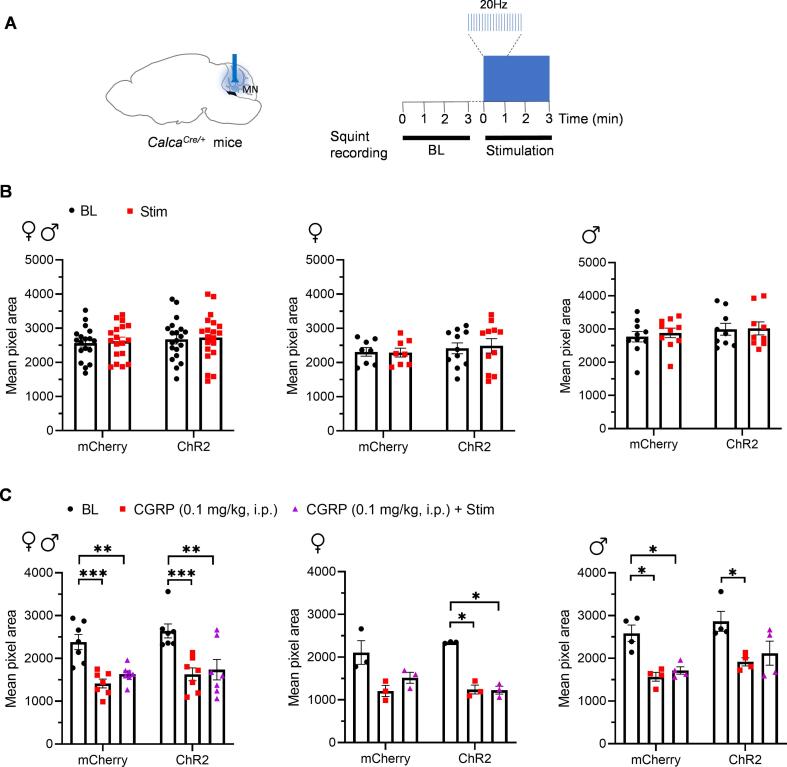
**Optical stimulation of MN^CGRP^ neurons did not induce nociceptive squinting behavior.** (A) Diagram representing the optogenetic stimulation strategy in the MN in *Calca^Cre/+^* mice during the automated squint assay (without CGRP treatment). Left panel: cre-dependent virus encoding either ChR2 or mCherry (control) was injected in the MN and the optical fibers were implanted above the MN. Right panel: experimental design. Baseline (BL) was first measured without stimulation. Immediately following the BL, mice were optically stimulated at 20 Hz with a 5 ms pulse width for 3 min during squinting recording. (B) Mean pixel area over 3-min testing period for individual mice without stimulation (BL) and with stimulation. The mean pixel area for all mice (left panel) (mCherry: n = 18; ChR2: n = 20), female mice (middle panel) (mCherry: n = 8; ChR2: n = 11), and male mice (right panel) (mCherry: n = 10; ChR2: n = 9). Data are from two independent experiments. (C) Mean pixel area over 3-min testing period for individual mice without stimulation (BL), i.p. 0.1 mg/kg CGRP only, or with stimulation and i.p. 0.1 mg/kg CGRP. The mean pixel area for all mice (left panel) (mCherry: n = 7; ChR2: n = 7), female mice (middle panel) (mCherry: n = 3; ChR2: n = 3), and male mice (right panel) (mCherry: n = 4; ChR2: n = 4). Data are from one experiment. For all panels, data are the mean ± SEM. Statistics are described in Supplementary Table 1. *P ≤ 0.05, **P ≤ 0.01, ***P ≤ 0.001.

Facial grimace has been used as an indicator of spontaneous pain in mice (Langford et al., 2010). A previous study by our laboratory demonstrated that squint is the principal component of the mouse grimace scale (Rea et al., 2018), and later an automated video-based squint assay was developed (Rea et al., 2021). This assay was able to measure spontaneous pain in a sensitive and objective manner. Thus, we intended to determine whether optical stimulation of MN^CGRP^ neurons can induce nociceptive squinting behavior. ChR2 and mCherry-expressing mice were optically stimulated with the protocol in Fig. 4A during the automated squint assay (10 mW power at the optical probe tip, 20 Hz, 5 ms pulse width, continuously for 3 min).

No difference in the mean pixel area was observed in the ChR2 or mCherry group with optical stimulation when comparing to respective baselines (Fig. 4B, left panel). This lack of an effect was observed in both female (Fig. 4B, middle panel) and male mice (Fig. 4B right panel). As a positive control to ensure that we could detect squint responses in these *Calca^Cre/+^* mice, we then tested the effect of i.p. CGRP (0.1 mg/kg) injections, which had previously been shown to induce squinting behavior detectable by the automated assay (Rea et al., 2021). Results showed that i.p. CGRP (0.1 mg/kg) significantly decreased the mean pixel area in both mCherry- and ChR2-expressing mice without stimulation in both sexes (Fig. 4C, left panel). When separated by sex, there was a significant difference or a decreasing trend in the mean pixel area between before and after i.p. CGRP (0.1 mg/kg) injection (Fig. 4C middle and right panels). The trending results may result from the small number of mice in each group. This finding suggests that these *Calca^Cre/+^* mice can squint under the condition of receiving i.p. CGRP (0.1 mg/kg). In addition, we are curious whether the mice need to be in a migraine-sensitized status for MN^CGRP^ neurons to induce a greater squint by combining i.p. CGRP and optical stimulation. We applied 0.1 mg/kg CGRP (i.p.), and a lower dose of CGRP (0.01 mg/kg, i.p.) as a control for a possible ceiling effect of 0.1 mg/kg CGRP (i.p.). However, no difference was detected between the combination and i.p. CGRP alone using both doses (Supplementary Fig. 3 and Fig. 4C), suggesting that the peripheral exogenous CGRP could not sensitize the MN^CGRP^ neurons to induce a greater squint. Thus, optical stimulation of MN^CGRP^ neurons did not induce nociceptive squinting behavior.

### 3.5 Optical stimulation of MN^CGRP^ did not induce gait alterations

**Fig. 5:**
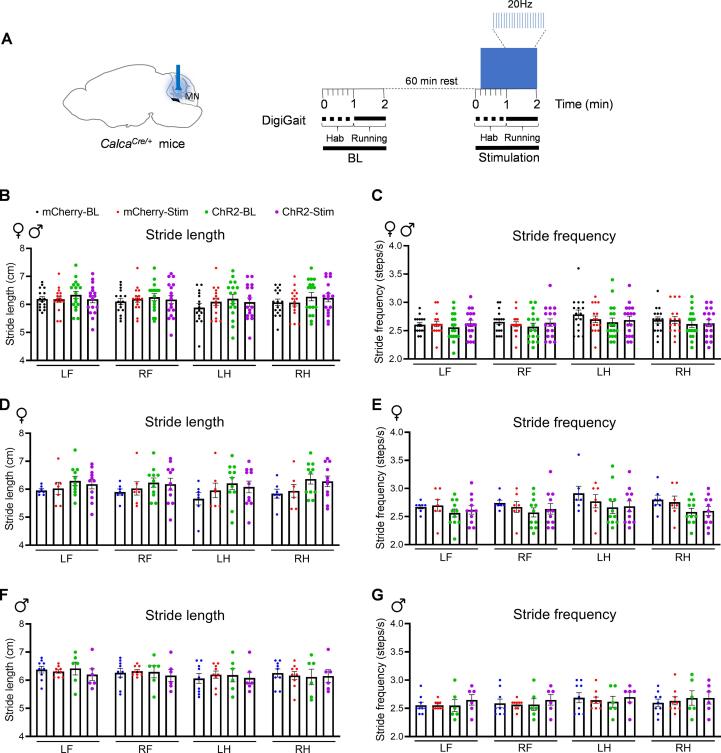
**Optical stimulation of MN^CGRP^ neurons did not induce gait alterations**. (A) Diagram representing optogenetic stimulation strategy in the MN in *Calca^Cre/+^* mice during the gait dynamic assay. Left panel: cre-dependent virus encoding either ChR2 or mCherry (control) was injected in the MN and the optical fibers were implanted above the MN. Right panel: experimental design. Mice were first habituated (Hab) for 1 min in the chamber before the baseline (BL) recording. After 60-min resting, mice were optically stimulated at 20 Hz with a 5 ms pulse width for 50 s before the treadmill was turned on and until the desired continuous running was obtained, approximately at a range of 60–110 s. (B-G) Stride length and frequency for all mice (B and C, mCherry: n = 16; ChR2: n = 17), female mice (D and E, mCherry: n = 7; ChR2: n = 11), and male mice (F and G, mCherry: n = 9; ChR2: n = 6). LF: left front paw; RF: right front paw; LH: left hind paw; RH: right hind paw. Data are from two independent experiments. For all panels, data are the mean ± SEM. Statistics are described in Supplementary Table 1.

The cerebellum is well known for its motor function, and the MN is responsible for controlling and maintaining posture and balance (Zhang et al., 2016). Therefore, we analyzed the gait dynamics using the DigiGait system. ChR2 and mCherry-expressing mice were optically stimulated with the protocol in Fig. 5A (10 mW power at the optical probe tip, 20 Hz, 5 ms pulse width). Mice were optically stimulated for 50 s before the start of the treadmill running and until the desired continuous running was obtained. There was no significant difference in either stride length or stride frequency between the baseline and stimulation in both mCherry and ChR2 groups compared to respective baselines across sexes (Fig. 5B and C) and within sexes (Fig. 5D-G). This finding indicates that optical stimulation of MN^CGRP^ neurons did not induce gait dysfunctions.

## 4 Discussion

Although CGRP is important in migraine pathophysiology (Goadsby et al., 1990, Ashina et al., 2000, Russo, 2019, Ashina et al., 2019, Edvinsson et al., 2018, Rapoport and McAllister, 2020) and CGRP is expressed in the MN (Warfvinge and Edvinsson, 2019), the role of these MN^CGRP^ neurons in migraine has not previously been explored. As a starting point, we showed that CGRP-expressing neurons in the MN project to regions implicated in photophobia, specifically multiple posterior thalamic nuclei. This agrees with reported connections between the MN and various thalamic nuclei (Fujita et al., 2020). We had previously observed CGRP fibers in posterior thalamic nuclei and found that CGRP injection into these nuclei induced light aversion (Sowers et al., 2020). In this study we have provided evidence that activation of cerebellar neurons causes migraine-like behaviors in mice. Most notably, optogenetic activation of MN^CGRP^ neurons caused light-aversive behavior only in female mice. In addition to this light sensitivity, touch sensitivity was also observed in both male and female mice, with the absence of observable motor deficits, anxiety, or spontaneous pain.

Photophobia is a subjective experience in which light is painful or uncomfortable and can cause or exacerbate headaches and is the most bothersome symptom other than pain (Noseda and Burstein, 2011, Munjal et al., 2020). One widely accepted model to rationalize the clinical manifestation of photophobia is the convergence of signals from intrinsically photosensitive retinal ganglion cells and nociceptive signals from the spinal trigeminal nucleus in the posterior thalamus (Noseda et al., 2019). Subsequently, these light and nociceptive signals are integrated in posterior thalamic neurons and sent to the somatosensory and visual cortices (Noseda et al., 2019). Evidence to support this model was reported by our laboratory; stimulation of the posterior thalamic region via CGRP direct administration or via optogenetic activation induced light aversion without accompanying anxiety-like responses (Sowers et al., 2020). In the current study, we conducted the light/dark assay to assess for light-aversive behavior, and the open field assay to assess the contribution of anxiety-like behavior to light aversion. We found that optical stimulation of MN^CGRP^ neurons induced light aversion without evoking anxiety-like behavior in female mice, suggesting a light-aversive response that was not solely driven by anxiety.

How might the cerebellum contribute to light-aversive behavior? One pathway may be projections from the MN to various thalamic nuclei, including parafascicular, centrolateral, mediodorsal, ventrolateral, suprageniculate, and posterior nuclei (Fujita et al., 2020). In this study, we showed that MN^CGRP^ neurons project to the posterior thalamic nuclei. In addition, the MN receives inputs from the spinal trigeminal nucleus via the cerebellar vermis, as well as inputs from the principle sensory trigeminal nucleus (Ge et al., 2014, Zhang et al., 2016). Thus, the MN lies in a circuit that receives signals from the trigeminal system and subsequently sends signals to the thalamus. In this circuit, the MN would be one source of CGRP in the posterior thalamic nuclei. In addition to posterior thalamic nuclei, MN^CGRP^ fibers were found in the nearby zona incerta. The zona incerta is of interest due to its inhibitory actions on the posterior thalamic nuclei through GABAergic projections (Bartho et al., 2002) and connections with all three deep cerebellar nuclei (Fujita et al., 2020, Teune et al., 2000,, Ossowska, 2020). While speculative, we propose that optical stimulation of MN^CGRP^ neurons may alter signaling in the posterior thalamic nuclei, leading to light-aversive behavior. Further studies are needed to measure neuronal activities of the thalamus as a result of optical stimulation of MN^CGRP^ neurons.

An open question is how might MN^CGRP^ neurons contribute to the observed female-specific light aversion? While the answer is not known, a sex difference in the cerebellum was reported in a human fMRI study (Maleki et al., 2012). That study exposed migraine patients to noxious stimuli and the results indicated that female migraine patients had higher cerebellar activation and greater deactivation of cerebellar functional connectivity with the insula than males did (Maleki et al., 2012). Moreover, whether there are sexually dimorphic differences regarding CGRP or CGRP receptors in the MN, and downstream brain regions remains to be investigated.

In addition to light aversion, we saw an allodynic response upon stimulating MN^CGRP^ neurons. There was a significant increase in sensitivity in response to mechanical stimuli in ipsilateral hind paws in both female and male mice. The results were less clear with the contralateral paw for female mice, but a response was observed in male mice. Allodynia is the perception of pain induced by non-noxious stimuli. Nearly 60% of individuals with migraine experience cutaneous allodynia, which is associated with migraine frequency, severity, and disability (Lipton et al., 2008, Bigal et al., 2008). Among these migraine patients, the most common form of allodynia is in the cephalic area, but approximately 25–50% of migraine patients report allodynia in the extracephalic area (Ashkenazi et al., 2007, Burstein et al., 2000, Guy, 2010, Mathew et al., 2004). Sensitization of second-order trigeminal and third-order thalamic neurons might result in the allodynia in cephalic and extracephalic areas in migraine patients (Burstein et al., 2000).

How might the cerebellum increase paw sensitivity bilaterally? There is evidence from preclinical and clinical studies that suggest a cerebellar role in affecting the descending pain modulation pathway (Saab et al., 2001, Hagains et al., 2011, Ruscheweyh et al., 2014, Dey and Ray, 1982), including via connections to the reticular formation bilaterally and the PAG contralaterally (Fujita et al., 2020, Teune et al., 2000,, Saab et al., 2001, Dey and Ray, 1982, Frontera et al., 2020). It also suggested that the MN modulates the dorsal column–medial lemniscus pathway directly (Saab et al., 2002). Furthermore, the MN likely plays a role in central sensitization and subsequent pain hypersensitive states based on evidence that the MN projects to the non-motor thalamic regions bilaterally (Fujita et al., 2020). It is interesting that we only observed evoked allodynia but not spontaneous, non-evoked squint pain responses with optical stimulation of MN^CGRP^ neurons. It suggests that sensory input (von Frey filament stimulation) is required, and stimulation is heightening the response to sensory input. The separation of these two phenotypes underscores the importance of measuring both evoked and spontaneous pain behaviors. Likewise, it was not surprising to observe that the lack of squint response did not match the light-aversive behavior since squint response is independent of light intensity (Rea et al., 2018).

There are several caveats for these behavioral studies. For the light aversion assay, both the ChR2 and control groups had a marked decrease in time in light over the 40 min testing period. While we usually see some decrease over time as mice start to lose exploratory drive (Mason et al., 2017, Kaiser et al., 2012), the decrease in this study was considerably greater than usual. We believe it is most likely due to the angle of the fiber-optic patch cord, which may have limited movement through the small opening between the light and dark zones. Wireless optogenetic approaches might solve this problem in the future. Another caveat is that we could not measure periorbital sensitivity in the tactile sensitivity test because we could not reproducibly habituate C57BL/6J mice (with or without surgeries) to low force von Frey filaments in periorbital areas. The reason for this is not clear since we can measure periorbital von Frey sensitivity in CD1 mice (Wattiez et al., 2021). Consequently, we were restricted to testing extracephalic (paw) sensitivity. Moreover, dural damage might possibly have a priming effect on mice behaviors. Furthermore, the unexplained contralateral hind paw sensitivity seen upon stimulation in female mice, even in the absence of ChR2, is a confounder that limits our ability to draw a firm conclusion about the female contralateral paw responses. The reason was unclear, but it might be due to the heat produced by the laser at the probe tips.

An additional caveat is that we used stimulation frequency at 20 Hz to stimulate MN^CGRP^ neurons, but we cannot be certain which neuronal types, excitatory and/or inhibitory, were targeted. There are multiple neuronal types in the MN (Fujita et al., 2020, Uusisaari et al., 2013). The majority of neurons in the MN are glutamatergic. There are also GABAergic neurons which are distributed sparsely and large glycinergic neurons. The spontaneous firing of these multiple neuronal types can range from less than 1 Hz to 30 Hz, while the maximum sustained firing can be more than 200 Hz (Uusisaari et al., 2013). Future experiments are needed to identify the neuronal types of these MN^CGRP^ neurons in *Calca^Cre/+^* mice, and it will be interesting to explore light aversion and tactile sensitivity using different laser frequencies.

**Fig. 6:**
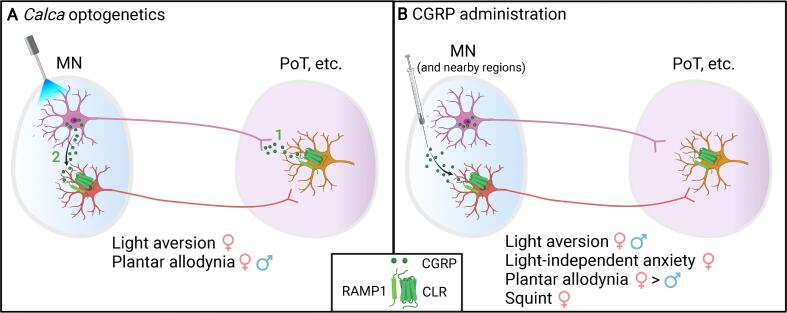
**Model of optical stimulation of MN^CGRP^ neurons and CGRP administration into the MN.** (A) With the optical stimulation, there are two sites of CGRP release. (1) MN^CGRP^ neurons release CGRP from axon terminals at the projection sites (e.g., PoT), where CGRP binds to CGRP receptors. (2) CGRP released from MN^CGRP^ neuron cell bodies binds to the CGRP receptors on other neurons in the MN that project to other brain regions (e.g. PoT). (B) With CGRP administration into the MN and diffusion into nearby regions, CGRP binds to CGRP receptors in the MN, which modulates CGRP receptors-expressing neurons and their projections to other brain regions (e.g. PoT). CLR: calcitonin receptor-like receptor; MN: medial cerebellar nucleus; PoT: posterior thalamic nuclei; RAMP1: Receptor activity-modifying protein 1. The figure was created with BioRender.com.

In conclusion, this study reveals that optical stimulation of CGRP-expressing neurons in the cerebellar MN is sufficient to induce two sensory phenotypes in mice that are surrogates for photophobia and cutaneous allodynia in humans. Specifically, stimulation of MN^CGRP^ neurons led to light aversion in female mice, and significant tactile hypersensitivity in ipsilateral hind paws in both sexes. A previous publication reported that direct injection of CGRP into the MN induced light aversion in both sexes, anxiety and squinting behavior in female mice, and more robust contralateral cutaneous allodynia in females than males (Wang et al., 2022). Interestingly, with both studies, the responses tended to be more predominant in female mice. The overlapping but distinct phenotypes following CGRP injection and optical stimulation of CGRP neurons is likely due to the targeting of different neuronal populations (Fig. 6). Optogenetic targeting was designed to activate CGRP-expressing neurons, including their projections to other brain regions, such as the posterior thalamic nuclei (Fig. 6A). In contrast, CGRP injection was designed to target CGRP receptor-expressing neurons, with the caveat that the injected peptide is capable of diffusing into neighboring areas (Fig. 6B). While, this proposed scenario shows two distinct targets (peptide vs receptor neurons), it is certainly possible that CGRP neurons in the MN may also express CGRP receptors, which may contribute to the shared phenotypes observed between the two approaches. Moving forward, colocalization of both the canonical CGRP and amylin type 1 (AMY_1_) receptors with CGRP should be examined. Overall, these results suggest that MN^CGRP^ neurons play a role in inducing migraine-like behaviors. Collectively, this study provides new insight into the increasingly complex neural circuitry underlying migraine.

### CRediT authorship contribution statement

**Mengya Wang:** Conceptualization, Methodology, Formal analysis, Investigation, Writing – original draft, Writing – review & editing, Visualization. **William C. Castonguay:** Investigation. **Thomas L. Duong:** Investigation, Writing – review & editing. **Michael W. Huebner:** Investigation, Formal analysis. **Harold C. Flinn:** Investigation. **Agatha M. Greenway:** Writing – review & editing. **Andrew F. Russo:** Conceptualization, Resources, Writing – review & editing, Visualization, Supervision, Project administration, Funding acquisition. **Levi P. Sowers:** Conceptualization, Methodology, Resources, Writing – review & editing, Visualization, Supervision, Project administration, Funding acquisition.

## Declaration of Competing Interest

The authors declare that they have no known competing financial interests or personal relationships that could have appeared to influence the work reported in this paper.
