# Supplementary material for: Stimulation of CGRP-expressing neurons in the medial cerebellar nucleus induces light and touch sensitivity in mice
Source: Neurobiol Pain. 2022 Jun 23;12:100098. doi: 10.1016/j.ynpai.2022.100098 (PMC9240374; doi:10.1016/j.ynpai.2022.100098)
Supplement: Supplementary data 1 [file mmc1.docx]

**
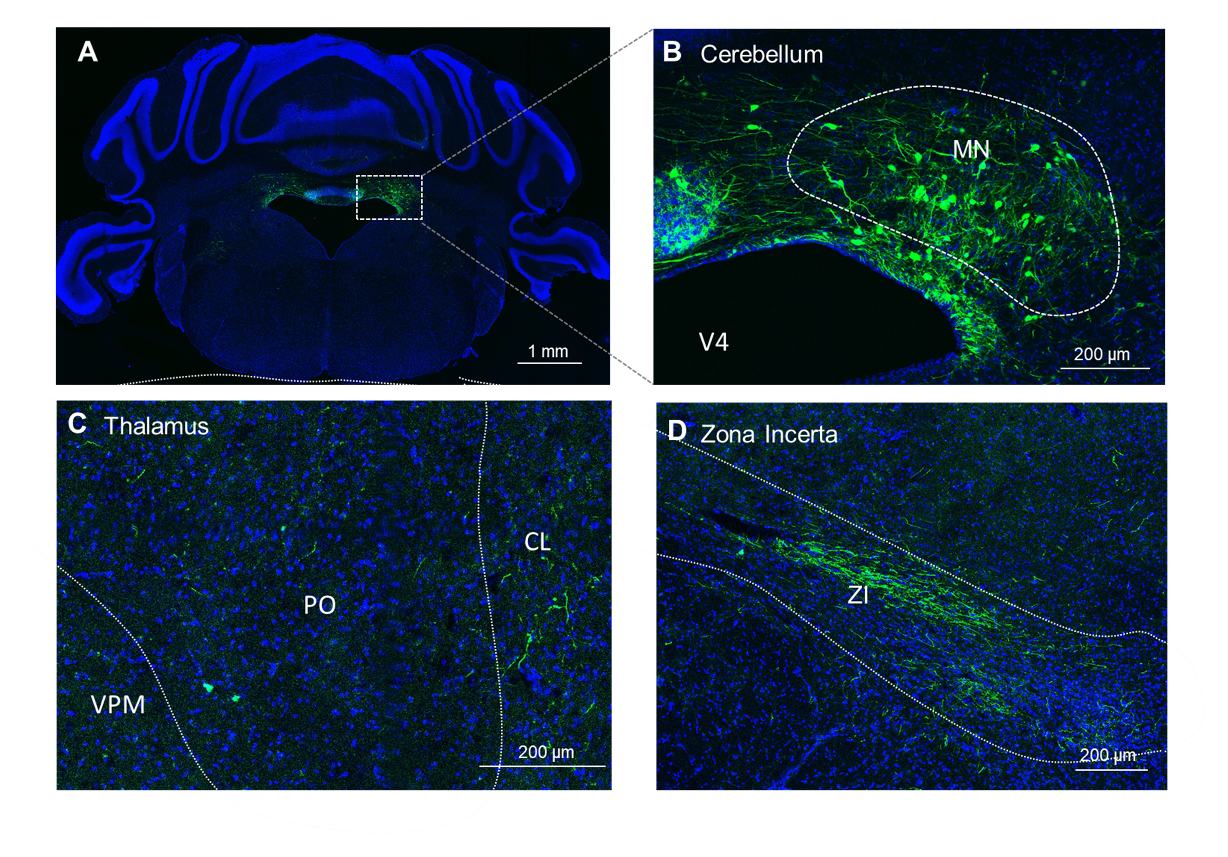
Supplementary materials**

**Supplementary Fig. 1. Validation of CGRP expression and projections in the MN of *Calca^Cre/+^* mice using AAV2-EF1a-DIO-EYFP virus.** (A) Representative example of a mouse with the expression of EYFP (green) in the MN after the injection of Cre-dependent virus AAV2-EF1a-DIO-EYFP into the MN. (B) A magnified image of the area within the rectangle in (A) showing EYFP-positive cell bodies and fibers in the MN (green, EYFP; blue, DAPI). (C and D) Representative examples showing fiber projections from the same mouse shown in (A). The EYFP fluorescence signals were found as fibers in posterior thalamic nuclei (C) and ZI (D). CL: central lateral nucleus of the thalamus; MN: medial cerebellar nucleus; PO: posterior complex of the thalamus; V4: fourth ventricle; VPM: ventral posteromedial nucleus of the thalamus; ZI: zona incerta.

**
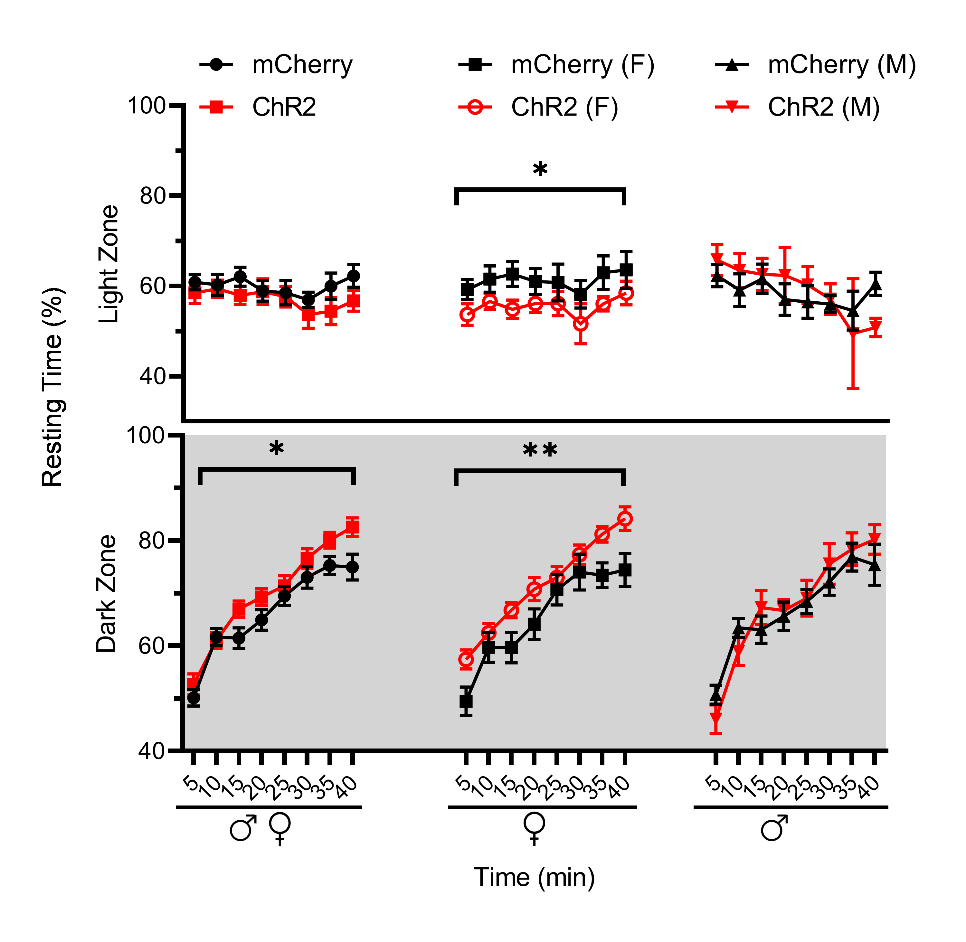
Supplementary Fig. 2. Optical stimulation of MN^CGRP^ neurons impacted motility.** Motility data were collected at the same time as light aversion data from the same mice shown in Fig. 2. Percentage of time spent resting in the light (upper panel) and dark (lower panel) zones every 5-min block during 40-min light/dark assay for all mice (left) (mCherry: n=19; ChR2: n=20), female mice (middle) (mCherry: n=9; ChR2: n=12), and male mice (right) (mCherry: n=10; ChR2: n=8). Data are from two independent experiments. Data are the mean ± SEM. Statistics are described in Supplementary Table 1.


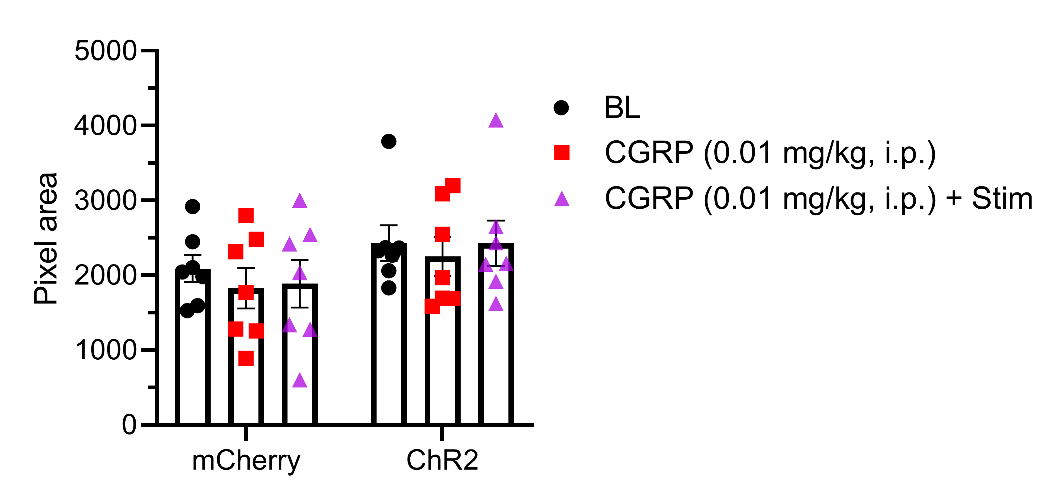


**Supplementary Fig. 3. Combination of low dose CGRP (0.01 mg/kg, i.p.) and optical stimulation of MN^CGRP^ neurons did not induce nociceptive squinting behavior.** Mean pixel area over 3-min testing period for individual mice without stimulation (BL), i.p. 0.01mg/kg CGRP only, or with stimulation and i.p. 0.01mg/kg CGRP. mCherry: n=7; ChR2: n=7. Data are from one experiment. Data are the mean ± SEM. Statistics are described in Supplementary Table 1.

**Supplementary Table 1. Statistical analyses**

| Fig. no. | Analysis | Statistics (symbol on Figure) | | | |
| --- | --- | --- | --- | --- | --- |
| Fig. 2B | | | | | |
| Left (all mice) | Two-way repeated measure ANOVA | | | | |
|  | Interaction factor | F (7, 259) = 0.7437, P=0.6351 | | | |
|  | Treatment factor | F (1, 37) = 8.997, P=0.0048 (**) | | | |
|  | Time factor | F (4.021, 148.8) = 58.47, P<0.0001 | | | |
| Middle (females) | Two-way repeated measure ANOVA | | | | |
|  | Interaction factor | F (7, 133) = 0.7651, P=0.6175 | | | |
|  | Treatment factor | F (1, 19) = 14.79, P=0.0011 (**) | | | |
|  | Time factor | F (4.608, 87.54) = 26.13, P<0.0001 | | | |
| Right (males) | Two-way repeated measure ANOVA | | | | |
|  | Interaction factor | F (7, 112) = 1.331, P=0.2423 | | | |
|  | Treatment factor | F (1, 16) = 0.2742, P=0.6077 | | | |
|  | Time factor | F (3.299, 52.78) = 36.15, P<0.0001 | | | |
| Fig. 2C | | | | | |
| Left (all mice) | Unpaired 2-tailed t-test | t=2.999, df=37, P=0.0048 (**) | | | |
| Middle (females) | Unpaired 2-tailed t-test | t=3.846, df=19, P=0.0011 (**) | | | |
| Right (males) | Unpaired 2-tailed t-test | t=0.9536, df=16, P=0.3545 | | | |
| ChR2-Female vs.ChR2-Male | Unpaired 2-tailed t-test | t=3.210, df=18, P=0.0049 (**) | | | |
| Fig. 2D |  |  | | | |
| Left (all mice) | Unpaired 2-tailed t-test | t=0.8039, df=37, P=0.4266 | | | |
| Middle (females) | Unpaired 2-tailed t-test | t=2.423, df=19, P=0.0255 (*) | | | |
| Right (males) | Unpaired 2-tailed t-test | t=0.7937, df=16, P=0.4390 | | | |
| ChR2-Female vs.ChR2-Male | Unpaired 2-tailed t-test | t=2.733, df=18, P=0.0137 (*) | | | |
| Fig. 2E | | | | | |
| Left (all mice) | Unpaired 2-tailed t-test | t=2.537, df=37, P=0.0155 (*) | | | |
| Middle (females) | Unpaired 2-tailed t-test | t=3.153, df=19, P=0.0052 (**) | | | |
| Right (males) | Unpaired 2-tailed t-test | t=0.3580, df=16, P=0.7250 | | | |
| ChR2-Female vs.ChR2-Male | Unpaired 2-tailed t-test | t=3.393, df=18, P=0.0032 (**) | | | |
| Fig. 2F | | | | | |
| Left (all mice) | Two-way repeated measure ANOVA | | | | |
|  | Interaction factor | F (7, 259) = 1.908, P=0.0687 | | | |
|  | Treatment factor | F (1, 37) = 2.086, P=0.1571 | | | |
|  | Time factor | F (7, 259) = 11.36, P<0.0001 | | | |
| Middle (females) | Two-way repeated measure ANOVA | | | | |
|  | Interaction factor | F (7, 133) = 3.508, P=0.0017 | | | |
|  | Treatment factor | F (1, 19) = 3.235, P=0.0880 | | | |
|  | Time factor | F (4.449, 84.52) = 6.319, P<0.0001 | | | |
|  | Šídák's multiple comparisons test | ns | | | |
| Right (males) | Two-way repeated measure ANOVA | | | | |
|  | Interaction factor | F (7, 112) = 0.4602, P=0.8614 | | | |
|  | Treatment factor | F (1, 16) = 7.401e-005, P=0.9932 | | | |
|  | Time factor | F (3.323, 53.16) = 5.576, P=0.0015 | | | |
| Fig. 2G | | | | | |
| Left (all mice) | Unpaired 2-tailed t-test | t=1.444, df=37, P=0.1572 | | | |
| Middle (females) | Unpaired 2-tailed t-test | t=1.799, df=19, P=0.0880 | | | |
| Right (males) | Unpaired 2-tailed t-test | t=0.008359, df=16, P=0.9934 | | | |
| ChR2-Female vs.ChR2-Male | Unpaired 2-tailed t-test | t=1.749, df=18, P=0.0973 | | | |
| Fig. 3B | | | | | |
| Left (all mice) | Two-way repeated measure ANOVA | | | | |
|  | Interaction factor | F (1, 37) = 11.44, P=0.0017 | | | |
|  | Treatment factor | F (1, 37) = 0.1943, P=0.6620 | | | |
|  | Condition factor | F (1, 37) = 23.54, P<0.0001 | | | |
|  | Paired 2-tailed t-test | ****P < .0001 | | | |
|  | Difference between BL and Stim: mCherry vs. ChR2 | | | | |
|  | Unpaired 2-tailed t-test | t=3.391, df=37, P=0.0017(**) | | | |
| Middle (females) | Two-way repeated measure ANOVA | | | | |
|  | Interaction factor | F (1, 19) = 5.144, P=0.0352 | | | |
|  | Treatment factor | F (1, 19) = 0.1586, P=0.6949 | | | |
|  | Condition factor | F (1, 19) = 20.59, P=0.0002 | | | |
|  | Paired 2-tailed t-test | ***P < .001 | | | |
|  | Difference between BL and Stim: mCherry vs. ChR2 | | | | |
|  | Unpaired 2-tailed t-test | t=3.636, df=19, P=0.0018(**) | | | |
| Right (males) | Two-way repeated measure ANOVA | | | | |
|  | Interaction factor | F (1, 16) = 4.968, P=0.0405 | | | |
|  | Treatment factor | F (1, 16) = 0.1165, P=0.7373 | | | |
|  | Condition factor | F (1, 16) = 5.249, P=0.0359 | | | |
|  | Paired 2-tailed t-test | ns | | | |
|  | Difference between BL and Stim: mCherry vs. ChR2 | | | | |
|  | Unpaired 2-tailed t-test | t=2.225, df=16, P=0.0408(*) | | | |
| Difference between BL and Stim in ChR2: Female vs. Male | | | | | |
|  | Unpaired 2-tailed t-test | t=0.8999, df=18, P=0.3801 | | | |
| Fig. 3C | | | | | |
| Left (all mice) | Two-way repeated measure ANOVA | | | | |
|  | Interaction factor | F (1, 38) = 4.269, P=0.0457 | | | |
|  | Treatment factor | F (1, 38) = 1.696, P=0.2006 | | | |
|  | Condition factor | F (1, 38) = 31.87, P<0.0001 | | | |
|  | Paired 2-tailed t-test | **P < .01, ****P < .0001 | | | |
|  | Difference between BL and Stim: mCherry vs. ChR2 | | | | |
|  | Unpaired 2-tailed t-test | t=1.524, df=38, P=0.1358 | | | |
| Middle (females) | Two-way repeated measure ANOVA | | | | |
|  | Interaction factor | F (1, 19) = 0.3167, P=0.5802 | | | |
|  | Treatment factor | F (1, 19) = 0.5820, P=0.4549 | | | |
|  | Condition factor | F (1, 19) = 15.38, P=0.0009 | | | |
|  | Paired 2-tailed t-test | *P < .05 | | | |
|  | Difference between BL and Stim: mCherry vs. ChR2 | | | | |
|  | Unpaired 2-tailed t-test | t=0.09027, df=19, P=0.9290 | | | |
| Right (males) | Two-way repeated measure ANOVA | | | | |
|  | Interaction factor | F (1, 17) = 8.883, P=0.0084 | | | |
|  | Treatment factor | F (1, 17) = 0.8808, P=0.3611 | | | |
|  | Condition factor | F (1, 17) = 21.62, P=0.0002 | | | |
|  | Paired 2-tailed t-test | **P < .01 | | | |
|  | Difference between BL and Stim: mCherry vs. ChR2 | | | | |
|  | Unpaired 2-tailed t-test | t=2.182, df=17, P=0.0434(*) | | | |
| Difference between BL and Stim in ChR2: Female vs. Male | | | | | |
|  | Unpaired 2-tailed t-test | t=1.084, df=19, P=0.2918 | | | |
| Fig. 4B | | | | | |
| Left (all mice) | Two-way repeated measure ANOVA | | | | |
|  | Interaction factor | F (1, 36) = 0.0002695, P=0.9870 | | | |
|  | Treatment factor | F (1, 36) = 0.3616, P=0.5514 | | | |
|  | Condition factor | F (1, 36) = 0.9181, P=0.3444 | | | |
| Middle (females) | Two-way repeated measure ANOVA | | | | |
|  | Interaction factor | F (1, 17) = 0.2170, P=0.6472 | | | |
|  | Treatment factor | F (1, 17) = 0.4526, P=0.5101 | | | |
|  | Condition factor | F (1, 17) = 0.06462, P=0.8024 | | | |
| Right (males) | Two-way repeated measure ANOVA | | | | |
|  | Interaction factor | F (1, 17) = 0.8442, P=0.3711 | | | |
|  | Treatment factor | F (1, 17) = 0.5916, P=0.4524 | | | |
|  | Condition factor | F (1, 17) = 2.079, P=0.1675 | | | |
| Fig. 4C | | | | | |
| Left (all mice) | Two-way repeated measure ANOVA | | | | |
|  | Interaction factor | F (2, 24) = 0.3091, P=0.7370 | | | |
|  | Treatment factor | F (1, 12) = 0.9917, P=0.3390 | | | |
|  | Condition factor | F (1.898, 22.78) = 56.01, P<0.0001 | | | |
|  | One-way repeated measure ANOVA | **P < .01, ***P < .001 | | | |
| Middle (females) | Two-way repeated measure ANOVA | | | | |
|  | Interaction factor | F (2, 8) = 2.342, P=0.1583 | | | |
|  | Treatment factor | F (1, 4) = 0.002781, P=0.9605 | | | |
|  | Condition factor | F (1.619, 6.476) = 38.96, P=0.0003 | | | |
|  | One-way repeated measure ANOVA | *P < .05 | | | |
| Right (males) | Two-way repeated measure ANOVA | | | | |
|  | Interaction factor | F (2, 12) = 0.08159, P=0.9222 | | | |
|  | Treatment factor | F (1, 6) = 3.480, P=0.1114 | | | |
|  | Condition factor | F (1.902, 11.41) = 23.02, P=0.0001 | | | |
|  | One-way repeated measure ANOVA | *P < .05 | | | |
| Fig. 5 |  | LF | RF | LH | RH |
| Fig. 5B (all mice) | Two-way repeated measure ANOVA | | | | |
|  | Interaction factor | F (1, 31) = 0.9728, P=0.3316 | F (1, 31) = 0.9143, P=0.3464 | F (1, 31) = 2.564, P=0.1195 | F (1, 31) = 0.04150, P=0.8399 |
|  | Treatment factor | F (1, 31) = 0.2651, P=0.6103 | F (1, 31) = 0.1911, P=0.6651 | F (1, 31) =0.6780, P=0.4166 | F (1, 31) = 1.157, P=0.2904 |
|  | Condition factor | F (1, 31) = 1.146, P=0.2927 | F (1, 31) = 0.0008396, P=0.9771 | F (1, 31) = 0.1919, P=0.6644 | F (1, 31) = 0.07652, P=0.7839 |
| Fig. 5C (all mice) | Two-way repeated measure ANOVA | | | | |
|  | Interaction factor | F (1, 31) = 0.6774, P=0.4168 | F (1, 31) = 1.658, P=0.2074 | F (1, 31) = 1.735, P=0.1974 | F (1, 31) = 0.02242, P=0.8820 |
|  | Treatment factor | F (1, 31) = 0.08575, P=0.7716 | F (1, 31) = 0.1768, P=0.6770 | F (1, 31) = 0.7632, P=0.3891 | F (1, 31) = 0.7246, P=0.4012 |
|  | Condition factor | F (1, 31) = 1.386, P=0.2481 | F (1, 31) = 0.09135, P=0.7645 | F (1, 31) = 0.2248, P=0.6387 | F (1, 31) = 0.02242, P=0.8820 |
| Fig. 5D (females) | Two-way repeated measure ANOVA | | | | |
|  | Interaction factor | F (1, 16) = 0.6892, P=0.4187 | F (1, 16) = 0.3749, P=0.5489 | F (1, 16) = 1.755, P=0.2039 | F (1, 16) = 0.4777, P=0.4994 |
|  | Treatment factor | F (1, 16) = 1.311, P=0.2690 | F (1, 16) = 1.063, P=0.3178 | F (1, 16) = 1.374, P=0.2582 | F (1, 16) = 3.001, P=0.1024 |
|  | Condition factor | F (1, 16) = 0.04190, P=0.8404 | F (1, 16) = 0.06127, P=0.8076 | F (1, 16) = 0.2867, P=0.5997 | F (1, 16) = 0.004777, P=0.9458 |
| Fig. 5E (females) | Two-way repeated measure ANOVA | | | | |
|  | Interaction factor | F (1, 16) = 0.05621, P=0.8156 | F (1, 16) = 0.8005, P=0.3842 | F (1, 16) = 1.036, P=0.3239 | F (1, 16) = 0.2467, P=0.6261 |
|  | Treatment factor | F (1, 16) = 0.9724, P=0.3388 | F (1, 16) = 1.030, P=0.3252 | F (1, 16) = 1.496, P=0.2389 | F (1, 16) = 3.502, P=0.0797 |
|  | Condition factor | F (1, 16) = 0.5755, P=0.4591 | F (1, 16) = 0.002664, P=0.9595 | F (1, 16) = 0.6209, P=0.4422 | F (1, 16) = 0.04032, P=0.8434 |
| Fig. 5F (males) | Two-way repeated measure ANOVA | | | | |
|  | Interaction factor | F (1, 13) = 0.5350, P=0.4775 | F (1, 13) = 0.8809, P=0.3651 | F (1, 13) = 0.7913, P=0.3899 | F (1, 13) = 0.2707, P=0.6116 |
|  | Treatment factor | F (1, 13) = 0.03876, P=0.8470 | F (1, 13) = 0.09548, P=0.7622 | F (1, 13) = 6.094e-029, P>0.9999 | F (1, 13) = 0.1038, P=0.7524 |
|  | Condition factor | F (1, 13) = 1.909, P=0.1904 | F (1, 13) = 0.1494, P=0.7054 | F (1, 13) = 0.01615, P=0.9008 | F (1, 13) = 0.05594, P=0.8167 |
| Fig. 5G (males) | Two-way repeated measure ANOVA | | | | |
|  | Interaction factor | F (1, 13) = 1.064, P=0.3212 | F (1, 13) = 1.175, P=0.2981 | F (1, 13) = 1.140, P=0.3052 | F (1, 13) = 0.1000, P=0.7568 |
|  | Treatment factor | F (1, 13) = 0.3009, P=0.5926 | F (1, 13) = 0.1072, P=0.7486 | F (1, 13) = 0.006605, P=0.9365 | F (1, 13) = 0.3364, P=0.5718 |
|  | Condition factor | F (1, 13) = 1.064, P=0.3212 | F (1, 13) = 0.3937, P=0.5412 | F (1, 13) = 0.1056, P=0.7504 | F (1, 13) = 0.1000, P=0.7568 |
| Supple. Fig. 2A upper panel | | | | | |
| Left (all mice) | Two-way repeated measure ANOVA (mixed effects analysis) | | | | |
|  | Interaction factor | F (7, 229) = 0.4969, P=0.8363 | | | |
|  | Treatment factor | F (1, 37) = 1.019, P=0.3193 | | | |
|  | Time factor | F (5.229, 171.1) = 0.7576, P=0.5871 | | | |
| Middle (females) | Two-way repeated measure ANOVA (mixed effects analysis) | | | | |
|  | Interaction factor | F (7, 124) = 0.1320, P=0.9958 | | | |
|  | Treatment factor | F (1, 19) = 5.911, P=0.0251 (*) | | | |
|  | Time factor | F (4.289, 75.98) = 0.8021, P=0.5351 | | | |
| Right (males) | Two-way repeated measure ANOVA (mixed effects analysis) | | | | |
|  | Interaction factor | F (7, 91) = 1.224, P=0.2980 | | | |
|  | Treatment factor | F (1, 16) = 0.1381, P=0.7150 | | | |
|  | Time factor | F (3.461, 44.99) = 1.923, P=0.1315 | | | |
| Supple. Fig. 2A lower panel | | | | | |
| Left (all mice) | Two-way repeated measure ANOVA (mixed effects analysis) | | | | |
|  | Interaction factor | F (7, 257) = 1.276, P=0.2623 | | | |
|  | Treatment factor | F (1, 37) = 5.899, P=0.0201 (*) | | | |
|  | Time factor | F (4.766, 175.0) = 69.00, P<0.0001 | | | |
| Middle (females) | Two-way repeated measure ANOVA (mixed effects analysis) | | | | |
|  | Interaction factor | F (7, 132) = 0.9004, P=0.5083 | | | |
|  | Treatment factor | F (1, 19) = 8.951, P=0.0075 (**) | | | |
|  | Time factor | F (4.380, 82.59) = 38.49, P<0.0001 | | | |
| Right (males) | Two-way repeated measure ANOVA (mixed effects analysis) | | | | |
|  | Interaction factor | F (7, 111) = 1.073, P=0.3853 | | | |
|  | Treatment factor | F (1, 16) = 0.1226, P=0.7308 | | | |
|  | Time factor | F (3.676, 58.28) = 30.72, P<0.0001 | | | |
| Supple. Fig. 3 | | | | | |
| All mice | Two-way repeated measure ANOVA (mixed effects analysis) | | | | |
|  | Interaction factor | F (2, 24) = 0.1810, P=0.8356 | | | |
|  | Treatment factor | F (1, 12) = 1.831, P=0.2009 | | | |
|  | Condition factor | F (1.922, 23.06) = 0.8602, P=0.4322 | | | |
